# Deprescribing: Reducing Harm, Enhancing Care

**DOI:** 10.5041/RMMJ.10575

**Published:** 2026-04-26

**Authors:** Carla Matos, Cindy Pinheiro

**Affiliations:** 1 Faculty of Health Sciences, Fernando Pessoa University, Porto, Portugal; 2 LAQV/REQUIMTE, Department of Chemical Sciences, Faculty of Pharmacy, University of Porto, Porto, Portugal; 3 RISE-Health, Faculty of Health Sciences, Fernando Pessoa University, Porto, Portugal

**Keywords:** Deprescribing, frail elderly, older people, polypharmacy

## Abstract

**Introduction:**

Polypharmacy carries the potential for increased risk and severity of adverse reactions. Deprescribing may reduce adverse events and possibly hospitalizations, although evidence on hard outcomes such as mortality is mixed. As the medication experts responsible for the close monitoring of their patient, doctors, nurses, and pharmacists play a crucial role in the process of medication deprescribing. They are involved in the therapeutic process and contribute to the appropriate use of medications and the proper discontinuation of those that no longer benefit the patient or are harmful. This role is particularly important for vulnerable groups, such as older people, who often take chronic medication.

**Objective:**

This study aims to review the literature on polypharmacy and the importance of suspending unnecessary or harmful medications in older people, emphasizing the role of healthcare professionals in the process.

**Methods:**

A narrative review of recent publications (articles from 2023 to 2025) in Web of Science, PubMed, and Scopus databases, addressing polypharmacy and deprescribing in older adults was conducted.

**Conclusions:**

A multidisciplinary team including doctors, nurses, and pharmacists plays a pivotal role in this process by reviewing patients’ therapeutic regimens, identifying inappropriate medications, and monitoring the withdrawal process. Their intervention can support more appropriate medication use, may improve patients’ quality of life, and has the potential to reduce healthcare costs.

## INTRODUCTION

The population of older adults has been increasing globally, and the population of octogenarians is expected to grow steadily.^1^ Aging involves a decline in the functional reserve of multiple organs and systems, resulting in altered pharmacokinetics. Hence, older adults experience increased comorbidities and reduced drug tolerance, thereby increasing the prevalence and severity of adverse effects.^2^ Furthermore, their increased comorbidities place older adults at significant risk of potentially inappropriate drug prescribing.^3^

Polypharmacy is a common condition that occurs when a person is prescribed multiple medications to treat different diseases or chronic symptoms.^4^ This can lead to a number of problems, including errors in taking or storing medicines, inappropriate prescriptions, drug interactions, and unwanted side effects.^3^,^4^ To prevent these problems, it is crucial to keep an accurate list of all prescribed medicines, including over-the-counter medications and supplements, and to periodically review this list with a doctor or pharmacist.^4^

Drug deprescribing is a practice for reducing the number of prescribed drugs that are no longer needed or that may be causing adverse effects.^5^,^6^ Healthcare professionals have a pivotal role in the deprescribing and implementation processes by reviewing medications and communicating with prescribers to adjust drug therapy. The aim is to optimize drug therapy and improve quality of life.^7^,^8^

This narrative review addresses the issue of polypharmacy in frail older adults, focusing particularly on certain therapeutic classes that are widely used by this age group. It also considers the role of healthcare professionals in identifying inappropriate medications and managing withdrawal.

## METHODS

A literature review was performed of scientific articles indexed in Web of Science, PubMed, and Scopus between January 2023 and May 2025, using the search terms “deprescribing” AND “elderly.” In addition to the initial search, we included documents identified through citation tracking and targeted manual searches, in order to clarify and densify specific information. All papers under consideration had to be full-text articles. The title and abstract of each article were screened. Articles included in this review had to be written in English, focus on the older adult (elderly) population, and address deprescribing or medication optimization. Studies were excluded if they involved non-elderly populations, were not directly related to the objectives of the review, were conference abstracts without full texts, or were duplicate records.

## RESULTS AND DISCUSSION

A total of 116 papers were identified for possible consideration. After applying the inclusion and exclusion criteria, 79 papers were selected. Key findings from the included studies were extracted and synthesized for this narrative review.

### Polypharmacy

The presence of two or more chronic health problems, known as multimorbidity, is quite common in older individuals and influences the therapeutic management carried out by both healthcare providers and patients.^9^,^10^ Because multimorbidity is highly prevalent in older adults and is more frequent in socioeconomically deprived populations, it has become a growing concern worldwide.^11^ The elderly population with multimorbidity often uses several medications to treat each condition; this is known as polypharmacy and is associated with negative outcomes such as mortality, falls, and adverse drug reactions.^10^,^12^,^13^

The literature has various definitions of polypharmacy, ranging from the use of two or more medications to eleven or more medications.^14^ However, according to Masnoon et al., the most common definition is the daily use of five or more medicines, a definition used by around 46.4% of studies.^10^ Some studies distinguish between the appropriate and inappropriate use of medicines. Appropriate polypharmacy is defined as optimizing the use of medicines in patients with complex and/or multiple conditions, in which the use of medicines is in line with the best available scientific evidence.^15^

Polypharmacy can be influenced by several factors, including the patient, the disease, the prescription, and accessibility to healthcare. Age is the main predictor of polypharmacy, with older age being positively correlated with the use of multiple medications. In addition, patient-related factors such as level of education, socioeconomic status, smoking, obesity, place of residence, and ethnicity may also be related to the risk of polypharmacy. In addition, the presence of chronic diseases is the main reason for polypharmacy. Finally, factors related to prescribing, such as changes in therapy and deprescribing, can also contribute to polypharmacy (Table 1).^16^

**Table 1:** Factors Associated with Polypharmacy.^16^

Patient-Related	Disease-Related	Prescribing-Related	Healthcare Accessibility-Related
Age Sex Level of education Socioeconomic status Smoking Obesity Place of residence Ethnicity Living as a couple Behavior	Chronic diseases: Cardiovascular Digestive Metabolic Respiratory	Physicians’ behavior Drug without an indication	Consulted a physician <3 mo. Hospitalization within last 6 mo. Health insurance Multiple providers

#### Epidemiology

The prevalence of polypharmacy can vary significantly, depending on various factors such as age group, healthcare setting, geographical region, and the definition used. Studies indicate that prevalence can range from 4% to 96.5%.^14^,^17^ A cross-sectional analysis of data from the Survey of Health, Aging, and Retirement in Europe (SHARE) database, which uses data from 17 European countries and includes Israel, shows that the prevalence of polypharmacy in adults over 65 varies between 26.3% and 39.9%. In Portugal, the overall prevalence of polypharmacy is 36.9% (95% CI 36.3–37.5).^18^

The European study Stimulating Innovation Management of Polypharmacy and Adherence in The Elderly (SIMPATHY) showed that economic deprivation has a significant impact on the prevalence of polypharmacy in all age groups, with higher rates among the most deprived groups. In the 65–69 age group, the prevalence of polypharmacy was 24% in the most deprived group, compared to just 7% in the least deprived group.^15^ It is important to emphasize that although the use of multiple medicines increases with age, men and women have almost identical rates of polypharmacy. Thus, polypharmacy is a problem that affects individuals of all age groups, but is particularly prevalent in older adults.^14^,^15^

Besides the demographic variations, the prevalence of polypharmacy is also strongly influenced by both clinical and healthcare system factors. The presence of multimorbidity appears as the primary determinant, leading to a higher treatment burden as medications are added to manage complex health needs.^14^,^16^ Additionally, the fragmentation of healthcare and management by multiple specialists without centralized coordination contribute to an increased number of prescriptions.^16^ Factors such as health literacy levels and geographic disparities between different national healthcare systems also explain why patients with similar clinical profiles may present significantly different therapeutic regimens.^17^,^18^

#### Clinical impact of polypharmacy

Polypharmacy in older adults is often linked to negative clinical outcomes, including frailty, hospitalization, falls, and mortality.^14^,^17^,^19^

Frailty is characterized by a state of non-resilience and increased vulnerability.^20^ Frail people are at greater risk of being prescribed extreme medication regimes, which can result in negative effects such as adverse reactions, drug interactions, and lack of adherence to treatment. However, more research is needed to clarify the relationship between polypharmacy and frailty syndromes and to identify ways to reduce medication burden and improve outcomes in this population.^20^–^22^

On the other hand, polypharmacy is related to higher rates of hospitalization, including those resulting from falls, incontinence, and delirium.^14^,^23^,^24^ According to Dhalwani et al., around one-third of the population who take five or more medications have a 21% increased risk of falling within two years.^23^ The number of medications used is independently associated with all-cause hospital admission, and excessive polypharmacy is a risk factor for returning to the emergency room and hospitalization within six months.^17^,^25^

Polypharmacy-related mortality risk is influenced by underlying chronic diseases,^26^ and the risk of death increases significantly as the number of medications rises.^14^,^17^ Specifically, categorized medication counts show a progressive increase in the pooled adjusted risk ratio (aRR) of death: 1.24 (95% CI 1.10–1.39) for 1–4 medications, 1.31 (95% CI 1.17–1.47) for 5 medications, and 1.59 (95% CI 1.36–1.87) for 6–9 medications. The risk is most pronounced in patients using 10 or more medications, with an associated mortality risk of 1.96 (95% CI 1.42–2.71). These results demonstrate the importance of a balanced approach to prescribing medicines in healthcare in order to reduce the risk of mortality associated with polypharmacy.^27^

#### Prescribing medicines for older adults

Treating older adults with medication is a major challenge, as they are more susceptible to adverse drug reactions due to changes in pharmacokinetics and pharmacodynamics.^28^ This issue becomes even more complicated in elderly people with neuro-cognitive disorders due to the high prevalence of multimorbidity, medication burden, and changes in neurotransmitters.^28^,^29^ Potentially inappropriate medications are those with greater risks than potential benefits, especially when other alternatives are available.^30^ The use of inappropriate medications in older adults has been a growing concern in the health field, as their use can cause adverse effects and reduce quality of life.^28^

Various tools are available for identifying potentially inappropriate medications. They should not only be used as a support to improve drug prescription, but also to measure and monitor the quality of drug treatment among older adults. It is important for healthcare professionals to take into account the possibility of adverse effects and to consider potentially inappropriate medicines when prescribing medicines for older adults, especially those with neuro-cognitive disorders.^28^

#### Pharmacological intervention tools

Pharmacological interventions, such as medication review, can reduce negative health outcomes and avoid excessive costs associated with prescribing potentially inappropriate medications. Several tools have been developed to detect potentially inappropriate prescriptions in older patients, including the Screening Tool of Older Persons’ Prescriptions (STOPP), the Screening Tool to Alert to Right Treatment (START) tools, the Beers criteria, and the Potentially Inappropriate Medication Check (PIM-Check) criteria.^31^

These instruments aim to improve the safety and quality of prescribing in older adult patients, who are often poly-medicated and at risk of adverse effects. Using them can help doctors and pharmacists make more informed and evidence-based decisions when prescribing medicines, reduce drug-related adverse effects, and improve quality of life.^31^ A recent umbrella review identified tools and guidelines to aid the deprescribing process of potentially inappropriate medications.^32^

##### (1) STOPP/START criteria

The STOPP/START criteria were developed to address the need for a deprescribing framework, providing structured guidance for identifying potentially inappropriate medications (STOPP) and omitted but indicated medications (START) in older adults.^33^–^37^ Each STOPP criterion is accompanied by a brief explanation of why the drug should be avoided in the older adult population, and reference is made to aspects related to dose, frequency, and duration of treatment. On the other hand, the START criteria refer to medicines that, according to the pathology of the older adult patient, have scientific evidence for safe prescription.^33^–^37^

Since their first publication in 2008, the STOPP and START criteria have been widely validated and translated into several languages, with a positive impact on patient assessment. The latest Version 3 (2023) includes 190 criteria (STOPP: 133; START: 57) based on updated clinical evidence and expert consensus.^33^

Studies applying these criteria have demonstrated their effectiveness in detecting inappropriate prescriptions and prescribing omissions that might otherwise be overlooked. The prevalence of patients with at least one occurrence of potentially inappropriate drug prescriptions identified by the STOPP criteria ranges from 21% among an Irish elderly population in primary care to 79% in the context of nursing home admissions in Spain.^34^ Use of the STOPP and START criteria has been associated with improved medication review processes, safer prescribing practices, and reduced medication-related harm, establishing them as validated and practical instruments for optimizing pharmacotherapy in older adults.^37^

Full access to clinical information is crucial for the correct application of the STOPP and START criteria. Only 29 of the STOPP criteria can be judged using only the patient’s current medication profile. The rest of the criteria require additional information, such as the duration of therapy, previous medication, current medical conditions, the patient’s medical history, laboratory data, and other measurable parameters. In the case of the START criteria, only one criterion can be judged based on current medication information alone.^38^

##### (2) Beers criteria

The aging of the world’s population has increased the burden of chronic disease and the long-term use of medication in older adults. Prolonged use of medicines in older adults has raised concern about adverse drug reactions and their public health impact. Inappropriate prescribing is an important contributor to these reactions and has led many countries to adopt standards of prescribing in older people, such as the Beers criteria.^39^

Developed by the American Geriatrics Society, the Beers criteria aim to improve drug selection and reduce adverse effects in adults aged 65 and over in all outpatient, acute, and institutionalized care settings. In 2019, the method was updated based on a comprehensive and systematic review and classification of evidence on drug-related problems and adverse effects in older adults. The strength and quality of each statement was graded based on the level of evidence and the strength of the recommendation.^40^ Overall, the criteria seek to improve the care of older adults by reducing their exposure to potentially inappropriate medications that have an unfavorable balance of benefits and harms compared to alternative treatment options. The Beers criteria can be used by practicing clinicians, as well as by patients, researchers, pharmacy benefit managers, regulators, and other stakeholders, to improve medication selection, educate clinicians and patients, reduce adverse drug effects, and assess the quality, cost, and utilization patterns of medications of older adults.^41^

##### (3) PIM-Check

The PIM-Check is an explicit, electronic prescribing tool developed to help clinical pharmacists and physicians detect potentially inappropriate prescriptions and other medication-related problems in adult internal medicine patients. It supports systematic medication reviews and the generation of targeted pharmaceutical interventions to optimize therapy and reduce avoidable adverse drug events.^42^–^44^

### Prescription of Drugs in Psychiatry

Mental illness affects one-third of the European Union’s population and represents a major burden on healthcare systems.^45^ Older people are particularly vulnerable to the adverse effects of psychotropic medicines due to age-related changes in pharmacokinetics and pharmacodynamics, high sensitivity to the effects of medication, and multiple comorbidities.^46^ Psychiatric disorders are more common in institutionalized older adults, and potentially inappropriate medication is a serious problem among hospitalized older people and residents of nursing homes.^47^ It is therefore necessary to incorporate deprescribing plans into routine care to mitigate these consequences, especially associated with high-risk medications such as antipsychotics, antidepressants, benzodiazepines, and others frequently used in people with dementia. Doctors require specific skills and knowledge in geriatric care, and geriatric assessment should include frequent review of prescribed and non-prescribed medications used by older adult patients.^48^

#### Benzodiazepines (BZD)

Benzodiazepines (BZD) were discovered accidentally in the 1950s and commercialized in 1963.^49^ They increase the effects of γ-aminobutyric acid, the main inhibitory neurotransmitter in the central nervous system, and are prescribed for a wide range of indications, including anxiety, sedation, and muscle relaxation.^49^,^50^ However, side effects such as cognitive impairment as well as medication abuse and dependence have been reported, especially in the older adult population.^49^,^50^ The inappropriate use of BZDs has become a growing public health problem as it can lead to drug-related hospital admissions.^50^,^51^

Nevertheless, BZDs are often prescribed to the older adult population. According to studies, the use of anxiolytics, hypnotics, and sedatives is more prevalent among elderly people aged between 85 and 89. Diazepam is the most frequently prescribed sedative, followed by alprazolam and oxazepam, while temazepam is the most frequently prescribed hypnotic, followed by nitrazepam.^46^

The elderly population is more sensitive to the serious side effects of BZDs, including falls and cognitive decline, due to age-related changes such as increased sensitivity of BZD receptors and the prolonged half-life.^46^,^47^,^50^ They also take more over-the-counter medicines, increasing the risk of harmful drug interactions and inadequate treatment.^50^ Misuse (an unfavorable balance of benefits and risks) and overuse (prescriptions that are not, or are no longer, necessary) often coexist with underuse of appropriate treatments, including underdiagnosis and failure to prescribe evidence-based medications for anxiety and sleep disorders when indicated.^50^

The misuse of BZD-related hypnotics in older adults represents an important issue for public health programs and healthcare professionals. The prevalence of various diseases increases with age, and understanding the epidemiology and definitions of BZD misuse in older adults is important for developing effective management strategies.^50^

Behavioral disorders are a common symptom of dementia, affecting 75% of institutionalized patients with the disease.^50^ Benzodiazepines have been used to treat these disorders, but their efficacy and safety are still debated.^52^ Studies suggest that BZDs can worsen symptoms and increase the risk of falls, delirium, respiratory failure, and car accidents.^47^,^50^ In addition, some studies suggest a possible association between BZD consumption and the risk of cancer or benign brain tumors. The acute adverse effects of BZDs on cognition have been well recognized, especially in the older adult population. Therefore, BZDs should be avoided in patients with Alzheimer’s disease, and clinicians should consider other therapeutic approaches, such as antidepressants.^50^–^52^ In addition, older adult patients should be appropriately warned about the dangers of BZD misuse, especially when combined with alcohol or opiates. In general, healthcare professionals should carefully weigh the risks and benefits of prescribing BZDs in patients with behavioral disorders associated with dementia, considering the clinical characteristics of each patient, their medication profile, and possible drug interactions.^50^

In conclusion, BZD use is widespread in elderly populations, particularly in care homes, and is associated with physical dependence, falls, and substantial economic burden.^53^ Careful monitoring and appropriate deprescribing strategies are therefore essential to ensure that BZDs are used only when benefits clearly outweigh the risks. Evidence from primary care settings suggests that BZD deprescribing is both feasible and can improve prescribing practices.^54^

### Antidepressants

The past 15 years has seen a significant increase in the use of antidepressants among older adults, both in the European Union and the United States. Studies have shown that almost 50% of nursing home residents are prescribed antidepressants.^55^,^56^ This increased prevalence is particularly marked in older people 75 years and over in community care homes. Furthermore, in long-term care homes, the proportion of residents receiving antidepressants for depression has increased.^56^ Comorbidities such as cardiovascular disease, anxiety disorders, arthritis, pain management, and osteoporosis are common in older people taking antidepressants, which can lead to inappropriate prescribing and increase the risk of adverse effects.^33^ Studies have shown frequent inappropriate use, both overuse and underuse, of antidepressants in older veterans living in community centers. However, there are disparities in antidepressant use between different age groups. Unmarried nursing home residents and those aged 85 or more are less likely to receive antidepressants when compared with married residents and those aged from 65 to 75 years, and Caucasians are more likely to receive antidepressants. In turn, the more mobile and independent residents, capable of decision-making, are less likely to receive antidepressants.^46^,^57^

Brisnik et al. developed and validated indicators signaling when a review of antidepressant therapy is important. They include both “high-risk” criteria (QTc prolongation, delirium, gastrointestinal bleeding, and liver injury risks in specific vulnerable patients) and “overprescribing” criteria (prolonged use, unsuitable indications such as mild depression or insomnia, or excessive dosing).^58^

### Prescription of Other Classes of Drugs

#### Non-steroidal anti-inflammatory drugs

Non-steroidal anti-inflammatory drugs (NSAIDs) are commonly prescribed to treat pain and musculoskeletal disorders, including arthritis. Their use increases with age, with 10%–40% of people over 65 using prescription or over-the-counter NSAIDs on a daily basis.^59^ Older adults frequently use NSAIDs, despite the significant risks of adverse effects, in-cluding gastrointestinal problems, kidney toxicity, fluid retention, and cardiovascular effects.^60^–^62^ Gastric protectants can help prevent some of these risks, but are often not prescribed with NSAIDs. Prescribers need to continually update their knowledge of potential interactions and safety signals to mitigate the risks. However, self-medication and the dispensing of non-prescription medicines are common practices.^60^,^61^

Nguyen et al. highlight the need for better methods to identify and select safe, effective medicines for older adults with multiple chronic conditions. Explicit prescribing tools and criteria-based lists, such as the STOPP and START criteria, the Beers criteria, and other NSAID-specific lists, are useful for safely prescribing NSAIDs in older adults. However, these lists should be applied together with a thorough assessment of each patient, including a risk–benefit analysis of any prescribed medication.^62^

Abdu et al. studied older patients (≥60 years) using NSAIDs and their associated risk factors, including polypharmacy and potential drug interactions. They showed that 20% of the study participants had polypharmacy, with diabetes and heart problems significantly associated with this outcome.^61^ In addition, only 25.4% of chronic NSAID users were co-prescribed gastro-protective agents, with omeprazole being the most frequently prescribed. Aspirin, ibuprofen, indomethacin, and diclofenac were the NSAIDs most frequently involved in potential drug interactions. Furthermore, as the number of drugs prescribed increased by one, the likelihood of interactions increased 3.25-fold. This risk was also higher among participants with diabetes or hypertension. The results of this study suggest that prescribers should be more vigilant in monitoring patients taking NSAIDs and consider gastro-protection when indicated.^61^

Hypertension is one of the most frequently diagnosed chronic conditions in older adults. These patients often receive both NSAIDs and antihypertensive drugs, which can have clinically significant drug interactions. The potential for this type of interaction should not be overlooked in clinical practice, as it may contribute to hypertension treatment failure in older patients. The degree to which blood pressure is influenced will depend on the type of antihypertensive drugs and NSAIDs used, with a smaller effect with calcium channel blockers.^60^

The assessment of the efficacy and toxicity of these drugs must consider the time it takes to reach the steady state. Most NSAIDs are well absorbed, showing minimal first-pass hepatic metabolism and binding tightly to serum proteins. The risk of adverse effects varies depending on the clinical context, medication, and dose. Interactions between NSAIDs and other drugs can increase the risk of toxicity. Several NSAIDs can increase the plasma levels of other drugs by partially inhibiting CYP-2C9 or CYP-2C8/2D6. As a result, acute and chronic renal alterations can be induced, including electrolyte acid–base disturbances, acute tubular necrosis, and acute interstitial nephritis, which can be accompanied by nephrotic syndrome and papillary necrosis.^59^

Olsen et al. discussed the use of non-vitamin K antagonist oral anticoagulants (NOACs) as an alternative to vitamin K antagonists (VKAs) for stroke prophylaxis in patients with atrial fibrillation (AF).^63^ They also examined gastrointestinal bleeding risk with concomitant NSAID use in AF patients receiving a VKA or NOAC. Although NOACs have similar or safer overall bleeding risk profiles compared to VKAs, some have been associated with significantly greater gastrointestinal bleeding than warfarin. The results of the study showed that the concomitant use of NSAIDs doubled the risk of gastrointestinal bleeding for NOACs and VKAs. Additionally, the authors showed that NOACs had lower overall gastrointestinal bleeding rates than VKAs, but the concomitant use of NSAIDs eliminated this advantage.^63^

Several studies discuss the effect of cyclooxygenase-1 and -2 inhibition on the development and recurrence of AF. They have shown that NSAIDs are associated with a higher risk of AF, especially in older adults and patients with chronic kidney disease or rheumatoid arthritis. In addition, a recent study showed that the consumption of NSAIDs, such as ibuprofen and diclofenac, increases the risk of cardiac arrest in patients who have suffered an out-of-hospital cardiac arrest.^59^ Indiscriminate use of NSAIDs can lead to deleterious side effects on the kidneys and heart.^62^ Alternative non-pharmacological therapies, such as exercise, weight loss, physiotherapy, and acupuncture, and pharmacological therapies such as paracetamol or medicinal plants, are effective options for treating pain. Although weak opioid drugs can be an effective option, the risk of addiction is a worldwide problem. Lifestyle modifications not only limit the use of NSAIDs but also provide a holistic approach.^64^

#### Proton pump inhibitors (PPIs)

Proton pump inhibitors (PPIs) are drugs used for gastrointestinal problems. However, a number of adverse effects associated with their prolonged use have been described, including cardiovascular disease,^65^ the development of dementia,^66^ *Clostridium difficile* infection, hip fractures, and pneumonia.^67^ Prescribing of these drugs has increased significantly in recent years, especially in older adults; however, several observational studies suggest that around two-thirds of patients receive inappropriate prescriptions without a gastrointestinal diagnosis.^66^,^68^

There is evidence of a relationship between PPI use and the development of adverse cardiovascular outcomes, with a hazard ratio of 2.11 for patients with more than 5.1 years of cumulative PPI exposure compared to individuals with no PPI exposure. Additionally, the hazard ratio of heart failure events is 2.21. Due to this association, Malik and Weintraub encourage the prompt discontinuation of PPIs when these drugs are no longer indicated.^65^

Gomm et al. found a significant association between PPI use and incident dementia, even after controlling for a variety of potential confounding factors.^66^ The use of PPIs was associated with a significantly increased risk of dementia (HR, 1.44 [95% CI, 1.36–1.52]; *P*<0.001) after adjusting for confounding factors such as age, gender, depression, stroke, diabetes, polypharmacy, and use of anticholinergic medications. The underlying mechanism by which PPIs may influence the development of dementia is still unclear, but there is evidence linking PPI intake to cognitive decline. Other studies have also reported that long-term PPI therapy may increase the fracture risk.^69^

The PPIs are an overused class of drugs that require deprescribing guidelines due to potential adverse effects and lack of continuous indication. Farrell et al. developed guidelines for healthcare professionals for reducing PPI use in patients who no longer need them. The guideline recommends short-term use of PPIs, advising clinicians to reduce PPI prescribing to the lowest effective dose before discontinuation, and to provide patients with a symptom management strategy that may include on-demand PPIs, or other over-the-counter agents or non-pharmacological approaches. Finally, the guideline suggests including a pharmacist in the interdisciplinary team to help reduce unnecessary PPI use and facilitate patient education, dose changes, and monitoring.^67^

All patients taking PPIs should have the indication regularly reviewed and clearly documented; those without a defining indication for chronic use should be considered for a deprescribing trial. In addition, most patients who require long-term PPI therapy but are taking twice-daily dosing should be considered for dose reduction to once daily.^68^,^70^

### Deprescribing

Deprescribing is a clinically supervised approach to stopping or reducing medications when they cause harm or no longer provide benefit.^5^,^6^

According to Alshatti et al., deprescribing can result in a reduction in mortality and the use of potentially inappropriate medications.^21^ Despite evidence that polypharmacy is associated with an increased risk of adverse outcomes in older adults, deprescribing has not been widely adopted, possibly because patients and providers are concerned about the effects of stopping medication, on health and on the therapeutic relationship. Efforts to develop and disseminate evidence-based guidelines could facilitate the wider adoption of deprescribing programs in healthcare systems.^21^

Nevertheless, deprescribing results in the literature are dubious. A recent systematic review and meta-analysis examined medication review and deprescribing in polypharmacy among hospitalized older adults. This meta-analysis of 30 studies showed an 8% reduction in hospital readmissions, while finding no significant impact on mortality. Only 16 studies showed significant benefits from deprescribing, mainly in measures of medication appropriateness, while 5 studies reported fewer adverse drug reactions; impacts on length of stay were inconsistent, and clinician uptake of deprescribing advice was often low.^71^ In a large, multinational cohort of multimorbid, poly-medicated older adults, discontinuing fall-risk-increasing drugs within two months of hospital discharge was common, yet one in four participants still fell during follow-up and no overall association was found between discontinuation of these drugs and subsequent falls. Notably, however, stopping antipsychotics was associated with a 68% reduction in fall risk among those with a prior fall.^72^

Another recent systematic review and meta-analysis of deprescribing studies states that withdrawing medications in older adults seldom translates into clear improvements in health outcomes such as mortality, falls, hospital use, or adverse drug withdrawal events—partly because those outcomes are multifactorial and may not shift when only one risk driver is modified. Class-specific effects varied: stopping antipsychotics or antihypertensives sometimes triggered symptom rebound, whereas patient-tailored medication reviews that included deprescribing showed modest survival gains when started early.^73^

Despite efforts to combat the inappropriate use of medication, there is no consensus on the best way to tackle the problem. A recent qualitative study examined the need to develop a clinical decision support system for drug deprescribing amongst physicians (*n*=10) in Sweden. Physicians in this study viewed deprescribing as inherently harder than prescribing, citing limited time, insufficient guidelines, fragmented patient care across multiple prescribers, and reluctance to alter specialists’ prescriptions; they also worried about managing taper plans and withdrawal symptoms. Patient-related hurdles—such as dependence, anxiety, and weak physician–patient trust—further complicate medication withdrawal.^74^

Across five Australian hospitals, surgical doctors and pharmacists reported only low-to-moderate confidence and capability in deprescribing, often misunderstanding it as simply withholding or rescheduling drugs around surgery. Gaps in knowledge, lack of clear guidelines, time pressures, heavy workloads, and a culture that does not prioritize medication withdrawal all undermine proactive practice.^75^

The Garfinkel Palliative-Geriatric method is a patient-centered deprescribing approach for frail older adults with multiple chronic conditions and limited life expectancy that seeks to minimize harm from inappropriate medication use and polypharmacy (see Table 2).^76^ In this model, as part of a comprehensive geriatric assessment, the benefits and risks of each medication are reviewed with the patient and family, and deprescribing as many non-life-saving drugs as possible is recommended, with regular follow-up. Medication deprescribing using this method has been associated with improvements in functional and cognitive status, sleep quality, appetite, sphincter control, quality of life, and overall satisfaction with care, as well as reduced number of serious complications, without increasing mortality or hospitalizations. However, reluctance among family doctors to adopt deprescribing recommendations remains a significant obstacle to wider implementation of this approach.^76^

**Table 2:** Understanding the Garfinkel Palliative-Geriatric Method.^76^

Pillars of the Garfinkel Palliative-Geriatric Method	
**Pillar 1**	**Vulnerable very old subpopulations with comorbidity, dementia, frailty, and limited life expectancy:** These patients often see many specialists who lack an integrative approach, and no single physician takes responsibility for the overall medication burden.
**Pillar 2**	**Limited trial evidence in older, frail populations:** Clinicians often extrapolate findings from randomized controlled trials in younger, healthier participants when prescribing for older, more vulnerable patients, which can lead to inappropriate clinical judgments and reduced quality of care.
**Pillar 3**	**The relationship between inappropriate medication use and polypharmacy:** The most effective solution is to reduce the overall drug count through deprescribing multiple medications.
**Clinical Application of the Garfinkel Palliative-Geriatric Method**	
**Step 1**	**Comprehensive geriatric assessment:** Evaluate the patient’s overall health.
**Step 2**	**Patient and family involvement:** Discuss the pros and cons of every drug with the patient or their family.
**Step 3**	**The Garfinkel algorithm:** Evaluate each medication following a specific decision-making algorithm. **Check evidence:** Is there evidence-based consensus for the drug’s indication in this specific age and disability group? Do benefits outweigh likely harms? **Validate indication:** Is the indication still relevant to the patient’s current health and disability level? If not, stop the drug. **Weigh risk/benefit:** Do known adverse reactions outweigh the possible benefits? If yes, switch to another drug. **Identify side effects:** Are there any current symptoms or signs possibly related to the drug? If yes, switch to another drug. **Consider alternatives:** Is there a superior drug available? If yes, switch to that drug. **Test dose reduction:** Can the dose be safely reduced? If yes, reduce the dose.
**Step 4**	**Implementation and follow-up:** Deprescribe as many non-life-saving drugs as possible and monitor outcomes for at least three months; follow up at least annually to assess clinical outcomes and satisfaction with care.

**Figure 1:**
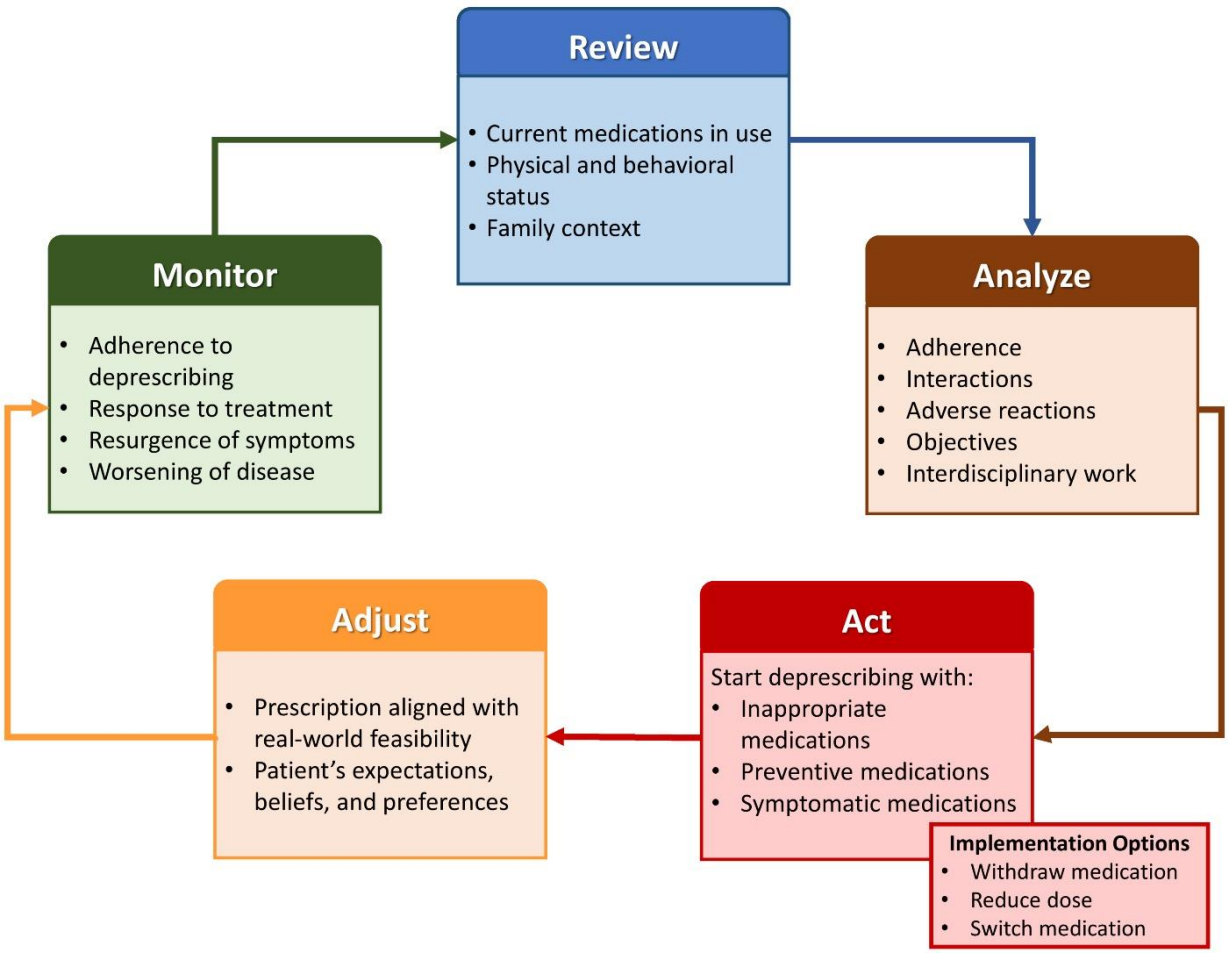
Steps in the Deprescribing Process. Adapted from Souza and Carneiro.^77^

Deprescribing should include the systematic identification of all medicines currently in use by the patient, to assess the benefits and harms of each medicine individually. According to Souza and Carneiro, the deprescribing process involves five steps, as detailed in Figure 1.^77^

On the other hand, deprescribing can also cause negative outcomes, such as adverse effects due to drug withdrawal, reappearance of medical conditions, reversal of drug interactions (for example, stopping an inducing or inhibiting drug and thereby increasing the toxicity of other medications), and consequences on the doctor–patient relationship. Adverse effects due to drug withdrawal are clinically significant symptoms caused by the removal of a drug and are frequent in older adult patients. These can be caused by the reappearance of the medical condition for which the drug was prescribed or due to a physiological reaction caused by the withdrawal of the drug. Physiological reactions can manifest as symptoms like those for which the drug is indicated or completely new symptoms. Examples of drugs that cause withdrawal effects are corticosteroids, drugs that act on the central nervous system, and gastrointestinal drugs. Weaning is recommended, rather than abrupt discontinuation, to avoid these negative effects; however, more studies are needed to better understand the regimens to be implemented.^5^

Another consequence of deprescribing is the reappearance of the initial symptoms, with a recurrence of the original condition. It can be difficult to differentiate between recurrence symptoms and adverse effects due to drug withdrawal; the timing of symptom onset can help to differentiate them. Deprescribing can damage the doctor–patient relationship, which is essential for providing patient-centered care and achieving the best health outcomes for individuals. Fear of negative consequences is a barrier to deprescribing. Patients are often seen as resistant to change and do not accept alternatives. In the context of end-of-life care, deprescribing preventative medication is seen as problematic, as it can be interpreted as withdrawal of care or giving up.^5^

Table 3 brings together a set of barriers and facilitators to deprescribing.

**Table 3:** Barriers and Facilitators of Deprescribing.^48^

Stakeholder	Selected Examples of Barriers and Facilitators to Deprescribing	
**Patient**	Barriers	Belief that medication is necessary and there will be negative results after medication withdrawal
	Facilitators	Desire to have a medication unprescribed
**Healthcare Professional**	Barriers	Belief that deprescribing would result in the onset of withdrawal symptoms or side effects Belief that deprescribing would be interpreted as a withdrawal of care Limited involvement of patients in the deprescribing decision
	Facilitators	Belief that the risk of continuing the medication outweighs the risk of deprescribing Awareness and trust in health professionals for the deprescribing of medications Involvement of patients in the deprescribing decision
**Healthcare Organization**	Barriers	Limited support for deprescribing interventions Limited resources and time to review drugs for deprescribing Prioritizing care of acute problems, neglecting appropriate prescribing for chronic diseases
	Facilitators	Organizational support and prioritization of multidisciplinary deprescribing interventions Adoption of computerized clinical decision support systems Adoption of quality improvement initiatives by healthcare professionals

In general, there is a lack of public knowledge and experience of deprescribing as a regular part of good care. Working in primary care is often a dichotomy when it comes to realizing deprescribing initiatives, and the relationship of trust that primary care professionals build with their patients can facilitate deprescribing approaches.^78^

In the context of secondary and residential care facilities, deprescribing is crucial, as studies show that there is an increase in the number of medications and the prevalence of potentially inappropriate medications during institutionalization. Despite the recommendations to avoid prescribing potentially inappropriate medications in older adults living in residential care facilities, studies show the high use of these medications, particularly important being the use of psychotropic medications, including antipsychotics, hypnotics, and anxiolytics.^78^

The European Union has set up a multidisciplinary and independent Expert Panel to advise on effective ways to invest in healthcare and has recently funded several projects that address the issue of polypharmacy and potentially inappropriate prescribing in older adults. These projects are based on collaborative and integrative approaches in different healthcare settings and emphasize working in partnership with patients to enable shared decision-making regarding medication.^48^

Deprescribing interventions can improve medication adherence measures in community-dwelling older adults who are largely independent, although they require considerable health literacy to manage the number, dosage, and complexity of their medications. However, in a study conducted by Ulley et al., it was shown that deprescribing as an intervention did not routinely improve medication adherence in the patient population studied, and further study in this area is recommended.^79^

## CONCLUSION

Polypharmacy is common in older adult patients with multimorbidities, making it a challenge for health management in these patients. The presence of multiple chronic conditions results in the prescription of multiple medications, which can result in adverse effects, decreased quality of life, increased hospitalizations, and increased mortality rates. On the other hand, polypharmacy is often associated with the prescription of potentially inappropriate medicines, which confer a greater risk of developing adverse effects or drug interactions, especially in older adults, due to factors associated with advanced age.

Deprescribing is a clinical approach to reducing or discontinuing the prescription of medicines that no longer have beneficial effects or are responsible for adverse effects, which can result in significant improvements to patients’ health. Effective medication review and deprescribing in older adults benefit from an interdisciplinary approach, involving physicians, pharmacists, and nurses. A patient-centered collaborative approach by healthcare professionals is essential for optimizing medication and mitigating the risks associated with polypharmacy. However, there are concerns and challenges associated with deprescribing, such as adverse effects due to drug withdrawal, reappearance of symptoms, reversal of drug interactions, and impact on the healthcare professional–patient relationship. It is important that health practitioners carefully consider the benefits and risks of deprescribing in each individual case and work in collaboration with patients and their families to make informed decisions.

## Abbreviations

AF: atrial fibrillation
BZD: benzodiazepines
NOAC: non-vitamin K antagonist oral anticoagulants
NSAIDs: non-steroidal anti-inflammatory drugs
PIM-Check: The Potentially Inappropriate Medication Checklist for Internal Medicine Patients
PPIs: proton pump inhibitors
SHARE: Survey of Health, Aging, and Retirement in Europe
SIMPATHY: Stimulating Innovation Management of Polypharmacy and Adherence in The Elderly study
START: Screening Tool to Alert to Right Treatment
STOPP: Screening Tool of Older Persons’ Prescriptions
VKAs: vitamin K antagonists
